# A comparison of meal tolerance test and oral glucose tolerance test for predicting insulin therapy in patients with gestational diabetes

**DOI:** 10.29219/fnr.v65.5490

**Published:** 2021-02-26

**Authors:** Mai Hijikata, Mariko Higa, Takamasa Ichijo, Takahisa Hirose

**Affiliations:** 1 Division of Diabetes, Metabolism, and Endocrinology, Department of Medicine, Toho University Graduate School of Medicine, Tokyo, Japan; 2 Division of Diabetes and Endocrinology, Department of Medicine, Saiseikai Yokohamashi Tobu Hospital, Kanagawa, Japan

**Keywords:** gestational diabetes, meal tolerance test, 75-g oral glucose tolerance test, insulin resistance, insulin therapy

## Abstract

**Aims:**

To identify factors predicting a need for insulin therapy in gestational diabetes mellitus (GDM) by comparing plasma glucose (PG) levels in a 75-g oral glucose tolerance test (75-g OGTT) with those in a 500-kcal meal tolerance test (MTT) containing 75 g of carbohydrate.

**Subjects and methods:**

The MTT was performed in 61 patients who diagnosed with GDM by a 75-g OGTT (age, 33.2 ± 4.5 years; prepregnancy body mass index, 22.6 ± 4.7 kg/m^2^; number of gestational weeks, 25.1 ± 6.4 weeks). PG and serum insulin levels were measured before the meal and up to 180 min after the meal. The insulin secretion capacity and resistance index were calculated.

**Results:**

PG levels increased from 86.8 ± 8.8 mg/dL at fasting to 132.7 ± 20.1 mg/dL at 30 min, and 137.8 ± 27.7 mg/dL at 60 min after MTT in the 35 patients with needed insulin therapy; these levels were significantly higher than those in the 26 patients, who only needed diet therapy. The patients with needed insulin therapy had significantly higher fasting PG levels in the 75-g OGTT, PG levels at fasting and 30 min after the MTT, and homeostasis model assessment of insulin resistance (HOMA-IR), and a significantly lower disposition index (DI) and insulin index than patients treated by diet alone. Receiver operating characteristic curve analysis was performed for factors involved in insulin therapy, with the following cutoff values: fasting PG in the 75-g OGTT, 92 mg/dL; PG 30 min after MTT, 129 mg/dL; HOMA-IR, 1.51; DI, 3.9; HbA1c, 5.4%. Multivariate analysis revealed that the 30-min PG level after MTT and HOMA-IR predicted insulin therapy.

**Conclusion:**

PG levels at 30 min after MTT may be useful for identifying patients with GDM, who need insulin therapy.

## Popular scientific summary

- This study aimed to compare a 75-g oral glucose tolerance test and a 500-kcal meal tolerance test (MTT) to identify factors predicting high-risk patients with gestational diabetes mellitus (GDM) who need insulin therapy.
- Plasma glucose at 30 min after MTT and homeostasis model assessment of insulin resistance may be useful for identifying patients at high-risk GDM.

In 2010, the International Association of Diabetes and Pregnancy Study Groups proposed new criteria for the diagnosis of gestational diabetes mellitus (GDM) (1) on the basis of Hyperglycemia and Adverse Pregnancy Outcome (HAPO) study (2). In the revised criteria, the cut-off values for fasting, 1-, and 2-h blood glucose levels in the 75-g oral glucose tolerance test (75-g OGTT) changed and if any of the blood glucose values are met or exceeded, a diagnosis of GDM is made (3). With the revised criteria, 17.8% of all pregnant women are now diagnosed with GDM, and the number of patients with GDM has increased almost fourfold from when the old criteria were used (1). Other reasons for an increase in the number of GDM may be an increase in the prevalence of overweight and inactivity and dietary changes (4–6). In the HAPO study, blood glucose levels in the 75-g OGTT before and 1 and 2 h after glucose intake were independently associated with maternal and perinatal complications (2). Another study found that GDM is also a risk for future disorders of glucose metabolism in the mother (7). The main pathogenic mechanism of GDM is an increase in maternal insulin resistance due to increased secretion of insulin-resistance hormones from the placenta, necessitating increased pancreatic β-cell insulin secretion (8–10). Although abnormal glucose tolerance in GDM was previously thought to be specific to pregnancy, the risk factors for GDM are now known to be the same as those for type 2 diabetes. Pregnancy has been equated to a stress test, in which placental growth hormones expose a mother’s predisposition toward metabolic disease (7, 9, 10). Strict blood glucose control is necessary to prevent maternal and fetal complications, and the first step is the dietary therapy and exercise. In many pregnant women, GDM can be managed with diet alone, but some high-risk patients require insulin therapy because of high blood glucose levels (11). It is important to establish in the early stages of GDM whether insulin therapy is required for glycemic control. However, it is difficult to predict on the basis of 75-g OGTT results alone whether GDM will require insulin therapy (11). One possible explanation for this difficulty is that the 75-g OGTT is a simple carbohydrate load, and the increase in the blood glucose level after the 75-g OGTT is not identical to daily plasma glucose (PG) fluctuations. Some studies have revealed factors that underlie a need for insulin therapy, but no consistent predictors have been found.

In this study, we conducted a 500-kcal meal tolerance test (MTT) to determine the PG levels, and the insulin secretion capacity and resistance index. We compared the results of the MTT and 75-g OGTT between the patients with high-risk GDM who require insulin therapy and the patients who was managed by diet alone, and we evaluated the efficacy of the MTT for predicting a need for insulin therapy. In addition, we noted any complications in the infants and compared prenatal and postnatal glucose tolerance in the mothers.

## Subjects and methods

This study included 61 patients with a diagnosis of GDM after a 75-g OGTT, who were admitted to our hospital for diet education and diabetic control between January 2018 and January 2019. The patients performed the 75-g OGTT because of a blood glucose level of 100 mg/dL or higher at any time in early pregnancy, a positive urine glucose, or a positive 50-g OGTT in midpregnancy, and they were diagnosed with GDM according to the new criteria (3). The 75-g OGTT at the time of diagnosis included only three-time measurements: immediately before, and 60 and 120 min after glucose consumption. We excluded patients who were started on insulin therapy before hospitalization or who had a diagnosis of diabetes before pregnancy and at the time of pregnancy. Patients with twins or who were taking ritodrine were also excluded. Patients with food allergies and gastro-intestinal disease were excluded. All participants provided a written informed consent.

In the MTT, after fasting overnight, patients ate a meal consisting of rice, scrambled eggs, braised radish, and yogurt (53% carbohydrate, 12% protein, and 25% fat), adjusted to 500 kcal and containing 75 g of carbohydrate, within approximately 15 min in the early morning on the second day of hospitalization. Blood samples were taken before the meal and 30, 60, 120, and 180 min after and used to assess PG and serum immune-reactive insulin (IRI) levels. The insulin resistance index was calculated from the homeostasis model assessment of insulin resistance (HOMA-IR) (fasting IRI [μU/mL] × fasting glucose [mg/dL]/405), and Matsuda index (calculated by using the online form available at http://mmatsuda.diabetes-smc.jp/MIndex.html) (12). The Matsuda index is an insulin sensitivity index derived from the ratio of PG to insulin concentration during an oral tolerance test (12, 13). Insulin secretion capacity index was calculated from the insulin index ([IRI 30 min after tolerance – fasting IRI]/[PG 30 min after tolerance – fasting PG]) and disposition index (insulin index × Matsuda index) (14). The area under the curve (AUC) for blood glucose and IRI over 120 and 180 min during the MTT and the AUC over 120 min for blood glucose during the 75-g OGTT were determined by the trapezoidal method (15). Clinical characteristics included age, number of weeks of gestation, body mass index (BMI) before pregnancy, weight gain until hospitalization, family history of diabetes in first- or second-degree relatives, and number of abnormal glucose values in the 75-g OGTT at the time of GDM diagnosis.

The diet after hospitalization was set at a total daily energy level of 30 kcal × standard body weight for overweight patients with a prepregnancy BMI ≥25 kg/m^2^. Patients with GDM who had a prepregnancy BMI below 25 kg/m^2^ were allowed to consume an additional 250 kcal regardless of the number of weeks of gestation. Patients whose postprandial blood glucose levels exceeded the target were given five or six divided meals. Blood glucose was measured by fingertip blood glucose measurement just before each meal and 2 h after each meal. Target blood glucose levels were 70–100 mg/dL before a meal and <120 mg/dL 2 h after. Insulin therapy was started in patients whose blood glucose exceeded the target levels at least twice. If premeal blood glucose levels exceeded the target, we administered insulin detemir (long-acting insulin), and if postprandial glucose levels exceeded the target, we administered insulin aspart (ultra-short-acting insulin). The insulin dose was increased by one to two units until target blood glucose levels were reached.

If the patients delivered in our hospital, we measured the birth weight and assessed for neonatal hypoglycemia. A 75-g OGTT was also performed in these patients 3 months after the delivery.

This study was conducted in accordance with the Declaration of Helsinki and was approved by our institutional review board (Saiseikai Yokohamashi Tobu Hospital Ethics Committee, approved on January 22, 2020, approval no. 20190117). In addition, the purpose and content of the study were fully explained in writing to the participants and their consent was obtained before the study was conducted.

### Statistical analysis

All data are presented as mean ± standard deviation (SD). To compare the MTT data over time, we used a repeated measures of analysis of variance (ANOVA) followed by Dunnett’s multiple comparison. Numerical data were compared between groups with a *t* test or Mann–Whitney *U* test. Pearson’s correlation coefficient was used for correlation analyses. We used receiver operating characteristic (ROC) analysis to analyze the factors involved in insulin therapy and to calculate the sensitivity and specificity with respect to each optimal cutoff. To identify predictive risk factors for insulin therapy, we performed a multivariate logistic regression analysis and calculated odds ratios with 95% confidence interval. In this study, two-sided *P* < 0.05 was considered significant. All statistical analyses were performed using SAS JMP version 11 (SAS Institute Inc., Cary, NC, USA).

## Results

### Patient background

The age of the patients studied was 33.2 ± 4.5 years; 24 patients were older than 35 years old and 37 were younger than 35 years. The prepregnancy BMI was 22.6 ± 4.7 kg/m^2^; 12 patients were overweight, with a prepregnancy BMI ≥27 kg/m^2^, and 49 were nonobese patients with a prepregnancy BMI of <27 kg/m^2^. Weight gain to hospitalization was 4.7 ± 3.4 kg (0–15 kg). The number of weeks of gestation was 25.1 ± 6.4 weeks (12–35 weeks): <24 weeks in 18 patients and ≥24 weeks in 43 patients. Thirty-one of the 61 patients had a family history of diabetes in first- or second-degree relatives. Glycated hemoglobin (HbA1c) was 5.3 ± 0.5%, and the number of abnormal glucose values in the 75-g OGTT at the time of GDM diagnosis was one in 34 patients, two in 17 patients, and three in 10 patients. Thirty-five of the 61 patients required insulin therapy, with a daily insulin requirement of 7.7 ± 11.4 units/day (3–49 units/day).

### Changes of PG and IRI levels during MTT in the insulin and diet therapy group

**Fig. 1:**
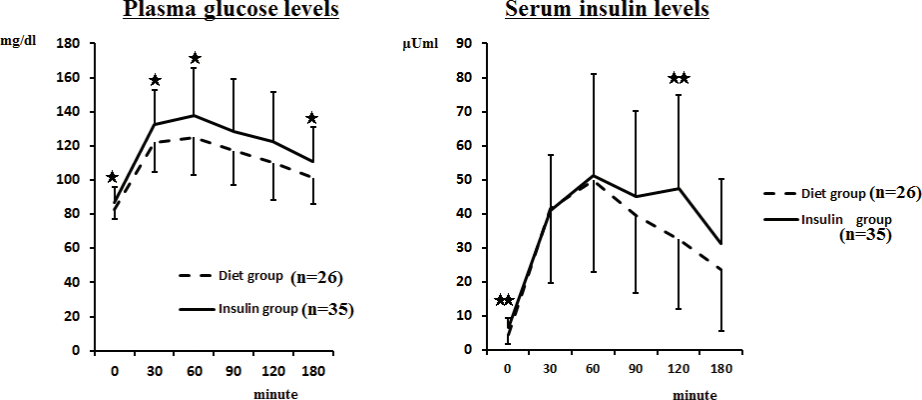
Changes in plasma glucose and serum insulin levels during meal tolerance test in the insulin therapy group and diet therapy group. Insulin therapy group vs. diet therapy group, *P < 0.05, **P < 0.01. Values are mean ± SD.

In the insulin therapy group, the PG level increased from 86.8 ± 8.8 mg/dL at fasting to 132.7 ± 20.1 mg/dL at 30 min and 137.8 ± 27.7 mg/dL at 60 min after the meal, which was the mean peak value. After 180 min, the level then decreased to 110.9 ± 20.0 mg/dL, but these values remained higher than the fasting PG level. In the diet therapy group, the PG level increased from 82.7 ± 6.0 mg/dL at fasting to 122.2 ± 17.8 mg/dL at 30 min and 125.0 ± 22.3 mg/dL at 60 min after the meal; these values were significantly lower (*P* < 0.05) than those in the insulin therapy group. The time to reach the peak PG level after the meal was 30 min in 23 patients, 60 min in 30 patients, and 90 min in eight patients. No significant differences were seen between the insulin and diet therapy groups in the time to reach the peak PG levels. Serum IRI levels at fasting and 120 min after a meal were significantly higher in insulin therapy group than those in the diet group (6.4 ± 2.8 vs. 4.6 ± 3.0 μU/mL at fasting, and 47.3 ± 27.7 vs. 32.6 ± 20.4 μU/mL at 120 min, *P* < 0.01) (Fig. 1).

### Association of MTT and 75-g OGTT at the time of GDM diagnosis

We examined whether the results of the MTT correlated with those of the 75-g OGTT performed at the time of diagnosis. In the insulin therapy group, the 120-min AUC for PG during the MTT showed significant positive correlations with the fasting, 1- and 2-h PG levels in the 75-g OGTT, and the 180-min AUC for PG during the MTT showed a positive correlation with the fasting and 1-h PG levels in the 75-g OGTT. Furthermore, the fasting PG in the 75-g OGTT was significantly positively correlated with HOMA-IR, and significantly negatively correlated with the Matsuda index, an index of insulin resistance. The insulin index and disposition index (DI) were not significantly related to 75-g OGTT PG levels. Meanwhile, in the diet therapy group, PG levels in the 75-g OGTT did not show a relationship with PG levels in the MTT, insulin resistance, or secretion index. We found no significant difference in insulin resistance or secretion capacity in the MTT between the patients diagnosed with GDM before (*n* = 18) and those diagnosed after (*n* = 43) 24 weeks of pregnancy (Table 1).

**Table 1:** Relationship between meal tolerance test and 75-g oral glucose tolerance test (OGTT) in the insulin therapy group

	Insulin therapy group *n* = 35			
	75-g OGTT			

	Fasting PG mg/dl	At 60 min PG mg/dl	At 120 min PG mg/dl	120-min PGAUC mg·min/dl
MTT				
120-min PGAUC (mg·min/dl)	No significant relationship (NS)	R = 0.48*	R = 0.38*	NS
180-min PGAUC (mg·min/dl)	R = 0.38*	R = 0.52*	NS	NS
HOMA-IR	R = 0.45*	NS	NS	NS
Matsuda Index	R = -0.46*	NS	NS	NS
Insulin Index	NS	NS	NS	NS
Disposition Index	R = -0.38*	NS	NS	NS

Note: 120 min PGAUC: area under the plasma glucose curve over 120 min; 180 min PGAUC: area under the plasma glucose curve over 180 min.

* *p* < 0.001, Values are mean ± SD.

### Risk analysis of factors affecting the requirement for insulin therapy

There were no significant differences in age, prepregnancy BMI, or number of weeks of gestation between the 35 patients on insulin therapy and the 26 patients on diet therapy. In contrast, the HbA1c level and fasting PG in the 75-g OGTT were significantly higher in the insulin therapy group than in the diet therapy group. No significant difference was seen in the positive number of abnormal values in the 75-g OGTT; however, insulin therapy was required in 9 of 10 patients with three abnormal values. The AUC values for PG over 120 and 180 min during the MTT showed no significant differences between the two groups. In the insulin therapy group, HOMA-IR was significantly higher than in the diet group, but the insulin index and DI were significantly lower (Tables 2).

**Table 2:** Comparison of clinical characteristics and metabolic indicators between insulin therapy group and diet therapy group

	Insulin group n = 35	Diet group n = 26	*P*
Age at GDM (years)	33.7±4.7	32.4±4.8	0.2861
Pre-pregnancy BMI (kg/m^2^)	23.3±3.8	22.7±4.6	0.5696
Number of week of gestation (week)	25.7±6.2	24.3±6.7	0.4139
Increase in body weight (kg)	5.21±3.40	4.06±3.40	0.1960
Family history of DM (+/-)	21/14	10/16	0.0948
HbA1c (%)	5.4±0.5	5.1±0.3	0.0164*
75-g oral glucose tolerance test (OGTT) Fasting plasma glucose (PG) (mg/dl)	95.4±8.5	90.1±10.8	0.0366*
75-g OGTT at 60 min PG (mg/dl)	179.1±41.1	171.3±29.8	0.4151
75-g OGTT at 120 min PG (mg/dl)	154.2±38.0	155.5±28.3	0.8872
75-g OGTT 120-min PGAUC (mg·min/dl)	1761.2±1107.8	1970.4±1066.1	0.4694
Number of abnormal glucose values in 75-g OGTT (points)	1.71±0.86	1.46±0.58	0.1766
Meal tolerance test (MTT) 120-min PGAUC (mg·min/dl)	4681.7±2089.2	3897.7±1726.4	0.1246
MTT 180-min PGAUC (mg·min/dl)	7192.3±3394.7	5889.2±2841.7	0.1180
MTT 180-min IRIAUC (μU·min/ml)	5134.1±2270.5	4407.8±1978.0	0.1937
HOMA-IR	1.41±0.68	0.96±0.68	0.0127*
Matsuda Index	7.65±5.3	9.78±5.0	0.1195
Insulin Index	0.70±0.66	1.00±0.48	0.0490*
Disposition Index	5.39±2.9	10.37±8.0	0.0051**

Note: 75-g OGTT 120-min PGAUC: area under the plasma glucose curve over 120 min during the 75-g OGTT; MTT 120-min PGAUC: area under the plasma glucose curve over 120 min during the MTT; MTT 180-min PGAUC: area under the plasma glucose curve over 180 min during MTT; MTT 180-min IRIAUC: area under the serum insulin curve over 180 min during MTT.

* *p* < 0.05

** *p* < 0.01. Values are mean ± SD.

**Fig. 2:**
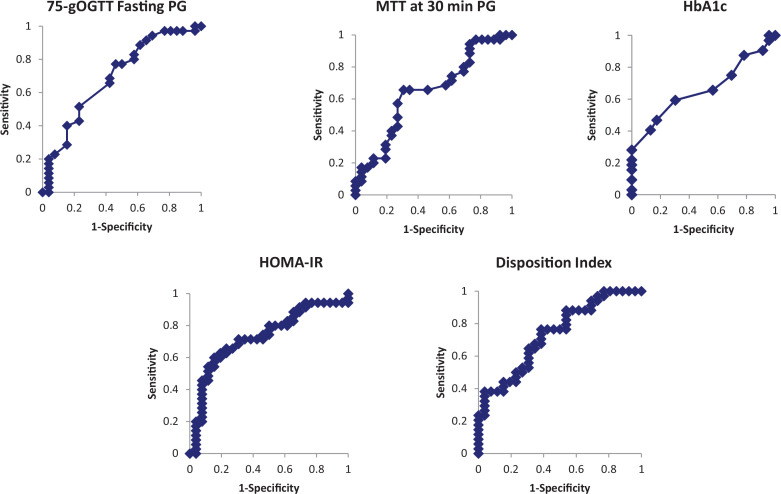
Receiver operating characteristics curve for fasting plasma glucose in 75-g OGTT, 30-min plasma glucose in MTT, HbA1c levels, HOMA-IR, and disposition index. OGTT: oral glucose tolerance test; PG: plasma glucose; MTT: meal tolerance test.

The ROC analysis of the factors involved in insulin therapy showed that the following parameters were significant (cutoffs in parentheses): OGTT fasting PG (92 mg/dL); MTT 30-min PG (129 mg/dL); HOMA-IR (1.51); DI (3.9); HbA1c (5.4%). The specificities of HOMA-IR, the DI, and HbA1c were high, but the sensitivities were low. In contrast, the sensitivities of 75-g OGTT fasting PG and MTT 30-min PG were high. The specificity was higher for MTT 30-min PG levels than for 75-g OGTT fasting PG levels with almost the same ROC areas (Table 3 and Fig. 2). To identify clinical factors that predict a need for insulin therapy, we performed multivariate logistic analysis for the five variables with a significant ROC areas, as shown in Table 3. Each variable was dichotically expressed according to the cutoff, as shown in Table 3. The 30-min PG in the MTT and HOMA-IR were identified by backward elimination methods as factors that predict a need for insulin therapy (Table 4).

**Table 3:** Receiver operating characteristics curve of risk factors identified as predictive of insulin therapy

	ROC		Optimal cut-off		
	AUC	p-value	Cut-off value	Sensitivity (%)	Specificity (%)
75-g OGTT Fasting PG (mg/dl)	0.693	0.0056**	92	77	54
MTT Fasting PG (mg/dl)	0.614	0.1153	90	31	92
MTT at 30 min PG (mg/dl)	0.646	0.0458*	129	66	69
MTT at 60 min PG (mg/dl)	0.632	0.0667	144	43	85
HOMA-IR	0.730	0.0006***	1.51	46	92
Insulin Index	0.630	0.0884	0.63	43	77
Disposition Index	0.735	0.0002***	3.90	38	96
HbA1c (%)	0.659	0.0296*	5.40	47	83

OGTT: oral glucose tolerance test; PG: plasma glucose; MTT: meal tolerance test; ROC: receiver operating characteristics; AUC: area under the curve.

* *p* < 0.05

** *p* < 0.01

*** *p* < 0.001

**Table 4:** Risk factors identified as predictive of insulin therapy in stepwise multiple logistic regression analyses

Dichotic variables	Odds ratio	95%CI	*p* value
1) All variables			
75-g OGTT Fasting PG	3.4584	0.9596–12.4682	0.0579
MTT at 30 min PG	3.9604	1.0251–15.3013	0.0459*
MTT at 60 min PG	0.7665	0.1672–3.5138	0.7321
Disposition Index	0.5026	0.0972–2.997	0.4119
HOMA-IR	3.6133	0.8946–14.5942	0.0713
2) Stepwise variable selection (backward elimination)			
MTT at 30 min PG	4.3537	1.2985-14.5974	0.0172**
HOMA-IR	5.4992	1.6582-18.2372	0.0053**

OGTT: oral glucose tolerance test; PG: plasma glucose, MTT: meal tolerance test.

* *p* < 0.05

** *p* < 0.01.

### Infant complications and postnatal OGTT results

Fifty-four patients delivered in our hospital. There was one case of hypoglycemia and one case of asphyxia in the neonates, but no malformations were found. Only one infant weighed more than 4,000 g. Within 3 months of delivery, the 75-g OGTT was performed in the 52 mothers; of these, 45 mothers had normal glucose tolerance (NGT), six had impaired glucose tolerance (IGT), and one mother was diagnosed with diabetes. We compared the PG levels at 30 min in the MTT performed before delivery between the 45 patients with NGT and the six patients with IGT and found that the PG levels in the MTT performed during pregnancy were significantly higher in the patients with IGT than in those with NGT (138.5 ± 35.2 mg/dL vs. 125.2 ± 15.6 mg/dL; *P* < 0.01). HOMA-IR from the MTT performed during pregnancy was significantly higher in the patients with IGT than in those with NGT (1.28 ± 0.70 vs. 1.12 ± 0.67; *P* < 0.05).

## Discussion

Glucose intolerance during pregnancy, even mild intolerance, is a risk for adverse perinatal outcome (2). The HAPO study found that the change in the cut-off value for blood glucose levels in the 75-g OGTT has led to an increase in the number of patients diagnosed with GDM (1, 3). Opinions on how to manage this increase vary from one institution to another; this is particularly true regarding how to deal with mildly impaired glucose intolerance in an OGTT result (16, 17). Physicians need to establish at the time of diagnosis of GDM whether insulin therapy is required for glycemic control. In previous studies, risk factors for insulin therapy in patients with three abnormal values in the OGTT results were similar to those identified in the present study (18). However, some patients were treated with insulin even if they had only one abnormal value in a 75-g OGTT result or mild glucose intolerance, making it difficult to identify high-risk GDM on the basis of OGTT results alone. This may be because the 75-g OGTT is a simple carbohydrate tolerance test, which is useful for detecting GDM, but the resulting blood glucose pattern differs from that observed during daily food intake (19). Therefore, in patients diagnosed with GDM, we compared the usefulness of MTT that included 75 g of carbohydrate and 75-g OGTT at a diagnosis of GDM in predicting the need for insulin therapy. We analyzed the risk factors for insulin therapy, including clinical parameters, blood glucose levels, and insulin resistance and secretion capacity. In almost all patients, the peak PG values during the MTT were at 30 or 60 min. PG levels during the MTT were well correlated with PG levels during the 75-g OGTT only in the insulin therapy group, however, these relationship were not observed in the diet therapy group. The reason for this discrepancy is unclear, but it might be due to a mild change of postprandial PG levels during the MTT in the diet therapy group (20). Furthermore, fasting PG levels in the 75-g OGTT correlated well with insulin resistance and secretion parameters only in the insulin therapy group.

In the ROC analysis, significant factors predictive of insulin therapy were fasting PG levels in the 75-g OGTT, PG levels 30 min after MTT, the HOMA-IR, DI, and HbA1c. The specificity of HOMA-IR, the DI, and HbA1c was high, but the sensitivity was low. In contrast, fasting PG levels in the 75-g OGTT and PG levels 30 min after MTT had low specificity and relatively high sensitivity. In the ROC analysis, the cut-off value for fasting PG in the 75-g OGTT was 92 mg/dL. Some previous reports showed that abnormal fasting PG levels in the 75-g OGTT are a risk factor for GDM and insulin therapy (21, 22). The present study also found that fasting PG levels in the 75-g OGTT were a risk factor for insulin therapy and fasting PG were correlated with insulin resistance. In this study, PG levels at 30 min after MTT might be selected for the risk factor need for insulin therapy though the mechanism of insulin resistance. The cut-off value for 30-min postprandial PG in the MTT was 129 mg/dL. The sensitivity of the 30-min postprandial PG level in the MTT was the same as the 75-g OGTT fasting glucose level; however, the specificity was relatively high, so the 30-min postprandial PG level in the MTT is considered to have high predictive validity for insulin therapy. Unfortunately, we are unable to compare 30-min postprandial PG level in the MTT with the 30-min glucose level in the 75-g OGTT, because we did not measure PG at 30 min in the 75-g OGTT. It is the limitation of this study. Together with HOMA-IR, the 30-min postprandial PG level in the MTT was selected as a risk factor for insulin therapy in the multivariate analysis. Some previous reports suggested that postprandial blood glucose levels predict the need for insulin therapy (19, 23). In addition, like the present study, some studies found that HbA1c, HOMA-IR, and the DI are predictors of insulin therapy (24).

Abnormal glucose tolerance in pregnancy is mainly caused by increased insulin resistance (9, 25) due to placenta-derived factors such as lactogen and prolactin, obesity, and inhibition of insulin signaling (9, 26). The pathogenesis of IGT in early pregnancy, when the placenta is still incomplete, may be different to that in mid-term and later pregnancy (25), but the details have not yet been elucidated. Insulin secretory capacity was found to increase during pregnancy to counteract increased insulin resistance, a process that may be important for maintaining NGT (27). This finding suggests that GDM can be used to predict not only specific metabolic abnormalities caused by pregnancy but also the patient’s future glucose tolerance of the patient (7, 9). Pancreatic β cells proliferate in the islets of Langerhans during pregnancy to increase insulin secretion in response to insulin resistance (28, 29). The relationship between insulin sensitivity and insulin secretion from pancreatic β cells is hyperbolic; therefore, if pancreatic β-cell function is equivalent, the product of insulin sensitivity and secretion (the DI) remains constant (30). However, in patients with IGT, the insulin sensitivity–secretion response curve is shifted to the left, and the DI is lower (30, 31, 32). In the present study, HOMA-IR was significantly higher and the DI was significantly lower in patients with GDM requiring insulin therapy than in those who managed their GDM with diet alone, which is consistent with previous reports (6, 24). In addition, the insulin index was also low in the group treated with insulin, suggesting that early insulin secretion capacity is reduced in high-risk GDM who require insulin therapy. This finding indicates that reduced insulin secretion capacity in response to insulin resistance is an important background factor in high-risk patients requiring insulin therapy. In this study, we found no significant differences in insulin resistance or secretion capacity between patients whose GDM was diagnosed in the first 24 weeks of pregnancy and those whose GDM was diagnosed later, and the study was unable to determine these pathologies in early pregnancies.

In the present study, the postprandial PG levels in the MTT peaked at 30 or 60 min and remained high, that is, they did not return to the premeal value even after 180 min. IRI also peaked at 60 min. The targets of blood glucose control during pregnancy are a fasting blood glucose below 95 mg/dL and a 2-h postprandial blood glucose below 120 mg/dL (33); however, in this study, the levels peaked at 30 or 60 min after the meal in the MTT. In recent years, a continuous glucose monitoring system has been developed to study blood glucose fluctuations in detail during pregnancy (34); however, an MTT is useful for assessing postprandial blood glucose levels and identifying high-risk patients requiring insulin therapy (19, 22). Further research is needed to determine whether the GDM control target for blood glucose 2 h after a meal is adequate. Furthermore, postprandial hyperglycemia is related to the amount of carbohydrates in a meal, and both a low-glycemic and a divided diet may be useful in controlling it (35).

During pregnancy, high PG levels at 30 min in an MTT and a high HOMA-IR may predict abnormal glucose tolerance in the 75-g OGTT after delivery. In particular, PG levels at 30 min in an MTT may not only predict high-risk GDM requiring insulin therapy but also contribute to predicting postpartum glucose intolerance. GDM is implicated in long-term maternal metabolic disorders and the development of cardiovascular disease (36, 37). The present results also suggest that insulin resistance and postprandial hyperglycemia in high-risk GDM patients may be risk factors for future metabolic disorders and cardiovascular disease.

## Conclusion

To examine the clinical background of pregnant women at high risk for requiring insulin therapy, 61 patients with GDM were given a 500-kcal MTT containing 75 g of carbohydrates. The results were compared with PG levels in the 75-g OGTT performed at the time of diagnosis. A need for insulin therapy was predicted by a fasting PG level of 92 mg/dL in the 75-g OGTT, a PG level of 129 mg/dL 30 min after the MTT, a HOMA-IR of 1.51, a DI of 3.9, and an HbA1c of 5.4%. In patients with GDM, a blood glucose level at 30 min after the MTT was predictive of IGT in a postpartum 75-g OGTT. The findings of this study had some limitations, which are as follows: (1) the number of patients was small; (2) the sample consisted of patients with GDM who required hospitalization; (3) the numbers of patients in the early and late stages of pregnancy were unequal; and (4) 30-min glucose levels were not measured in the 75-g OGTT, so these values could not be compared with the 30-min postprandial PG level in the MTT. Further studies in large samples will be necessary in future.

## References

[1] International Association of Diabetes and Pregnancy Study groups. International Association of Diabetes and Pregnancy Study groups recommendations on the diagnosis and classification of hyperglycemia in pregnancy. Diabetes Care 2010; 33(3): 676–82. 20190296 10.2337/dc09-1848 PMC2827530

[2] HAPO study cooperative research group. Hyperglycemia and adverse pregnancy outcomes. N Engl J Med 2008; 358: 1991–2002. 18463375 10.1056/NEJMoa0707943

[3] Coustan DR. Gestational diabetes mellitus. Clinical Chemistry 2013; 59: 1310–21. 23536513 10.1373/clinchem.2013.203331

[4] Machado C, Monteiro S, Oliveira MJ. Impact of overweight and obesity on pregnancy outcomes in women with gestational diabetes – Results from a retrospective multicenter study. Arch Endocrinol Metab 2020; 64(1): 45–51. 31576966 10.20945/2359-3997000000178 PMC10522280

[5] Alves JG, Souza ASR, Figueiroa JN, Araūjo CAL, Guimaraes A, Ray JG. Visceral adipose tissue depth in early pregnancy and gestational diabetes mellitus – A cohort study. Sci Rep 2020; 10: 2032. 32029868 10.1038/s41598-020-59065-5 PMC7005273

[6] Sun YY, Juan J, Xu QQ, Su RN, Hirst JE, Yang, H-X. Increasing insulin resistance predicts adverse pregnancy outcomes in women with gestational diabetes mellitus. J Diabetes 2020; 12(6): 1–9. 10.1111/1753-0407.13013 31808991

[7] Bellamy L, Casas J-P, Hingorani AD, Williams D. Type 2 diabetes mellitus after gestational diabetes: a systematic review and meta-analysis. Lancet 2009; 373: 1773–9. 19465232 10.1016/S0140-6736(09)60731-5

[8] Nadal A, Alonso-Magdalena P, Soriano S, Ropero AB, Quesada I. The role of oestrogens in the adaptation of islets to insulin resistance. J Physiol 2009; 587(21): 5031–7. 19687125 10.1113/jphysiol.2009.177188 PMC2790246

[9] Barbour LA. Metabolic culprits in obese pregnancies and gestational diabetes mellitus: big babies, big twists, big picture. Diabetes Care 2019; 42: 718–26. 31010942 10.2337/dci18-0048 PMC6489109

[10] Buchanan TA. Pancreatic b-cell defects in gestational diabetes: implications for the pathogenesis and prevention of type 2 diabetes. J Clin Endocrinol Metab 2001; 86(3): 989–993. 11238474 10.1210/jcem.86.3.7339

[11] Papachatzopoulou E, Chatzakis C, Lambrinoudaki I, Panoulis K, Dinas K, et al. Abnormal fasting, post-load or combined glucose values on oral glucose tolerance test and pregnancy outcomes in women with gestational diabetes mellitus. Diabetes Res Clin Pract 2020; 161: 108048. 32027925 10.1016/j.diabres.2020.108048

[12] Matsda M, DeFronzo RA. Insulin sensitivity indices obtained from oral glucose tolerance testing: comparison with euglycemic insulin clamp. Diabetes Care 1999; 22(9): 1462–70. 10480510 10.2337/diacare.22.9.1462

[13] Henríquez S, Jara N, Bunout D, Maza SHMP, Leiva L, Barrera G. Variability of formulas to assess insulin sensitivity and their association with the Matsuda index. Nutr Hosp 2013; 28(5): 1594–8. 24160221 10.3305/nh.2013.28.5.6512

[14] Yokota K, Fukushima M, Takahashi Y, Igaki N, Seino S. Insulin secretion and computed tomography values of pancreas in the early stage of the development of diabetes. J Diab Invest 2012; 3(4): 371–6. 10.1111/j.2040-1124.2012.00212.x PMC4019257 24843592

[15] Wilkins PA, Sheahan BJ, Vader Werf KA, Castagnetti C, Hardy J, Schoster A, et al. Preliminary investigation of the area under the L-lactate concentration-time curve (LAC AREA) in critically Ill equine neonates. J Vet Intern Med 2015; 29: 659–662. 25818220 10.1111/jvim.12559 PMC4895514

[16] Ikenoue S, Miyakoshi K, Saisho Y, Sakai K, Kasuga K, Fukutake M, et al. Clinical impact of women with gestational diabetes mellitus by the new consensus criteria: two year experience in a single institution in Japan. Endocr J 2014; 61(4): 353–8. 24430729 10.1507/endocrj.ej13-0496

[17] Sugiyama T, Metoki H, Hamada H, Nishigori H, Saito M, et al. A retrospective multi-institutional study of treatment for mild gestational diabetes in Japan. Diabetes Res Clin Pract 2014; 103(3): 412–18. 24485857 10.1016/j.diabres.2013.12.017

[18] Nishikawa T, Ono K, Hashimoto S, Kinoshita H, Watanabe T, Araki H, et al. One-hour oral glucose tolerance test plasma glucose at gestational diabetes diagnosis is a common predictor of the need for insulin therapy in pregnancy and postpartum impaired glucose tolerance. J Diabetes Invest 2018; 9: 1370–7. 10.1111/jdi.12848 PMC6215946 29624902

[19] Marais C, Hall DR, Wyk LV, Conradie M. Randomized cross-over trial comparing the diagnosis of gestational diabetes by oral glucose tolerance test and a designed breakfast glucose profile. Inter J Gynecol Obstet 2011; 141(1): 85–90. 10.1002/ijgo.12427 29243247

[20] Cheney C, Shiragg P, Hollingsworth D. Demonstration of heterogeneity in gestational diabetes by a 400-kcal breakfast meal tolerance test. Obstet Gynecol 1985; 65(1): 17–23. 3880878

[21] Hao M, Lin L. Fasting plasma glucose and body mass index during the first trimester of pregnancy as predictors of gestational diabetes mellitus in a Chinese population. Endocr J 2017; 64(5): 561–9. 28420856 10.1507/endocrj.EJ16-0359

[22] Jokelainen M, Lempinen BS, Rȍnȍ K, Nenonen A, Kautiainen H, et al. Oral glucose tolerance test results in early pregnancy: a Finnish population-based cohort study. Diabetes Res Clin Pract 2020; 162: 108077. 32057964 10.1016/j.diabres.2020.108077

[23] Sharma K, Wahi P, Gupta A, Jandial K, Bhagat R, et al. Single glucose challenge test procedure for diagnosis of gestational diabetes mellitus: a Jammu cohort study. J Assoc Physicians India 2013; 61(8): 558–9. 24818340

[24] Tang L, Xu S, Li P, Li L. Predictors of insulin treatment during pregnancy and abnormal postpartum glucose metabolism in patients with gestational diabetes mellitus. Diabetes Metb Syndr Obes 2019; 12: 2655–65. 10.2147/DMSO.S233554 PMC6914658 31853192

[25] Catalano PM, Tyzbir ED, Roman NM, Amini SB, Sims EA. Longitudinal changes in insulin release and insulin resistance in nonobese pregnant women. Am J Obstet Gynecol 1991; 165: 1667–72. 1750458 10.1016/0002-9378(91)90012-g

[26] Wang L, Hao JM, Yu AQ, Li TT, Liu RR, Li J, et al. The association of plasma peroxiredoxin 3 with insulin in pregnant women. Biochem Biophys Res Commun 2019; 508(3): 805–10. 30528738 10.1016/j.bbrc.2018.12.021

[27] Moyce BL, Doinsky VW. Maternal β-cell adaptations in pregnancy and placental signalling: implication for gestational diabetes. Int J Mol Sci 2018; 19: 3467. 10.3390/ijms19113467 PMC6274918 30400566

[28] Sorenson RL, Brelje TC. Adaptation of islets of Langerhans to pregnancy: beta-cell growth, enhanced insulin secretion and the role of lactogenic hormones. Horm Metab Res 1997; 29(6): 301–7. 9230352 10.1055/s-2007-979040

[29] Yang W, Jiang Y, Wang Y, Zhang T, Liu Q, Wang C, et al. Placental growth factor in beta cells plays an essential role in gestational beta-cell growth. BMJ Open Diab Res Care 2020; 8: e000921. 10.1136/bmjdrc-2019-000921 PMC7059504 32144129

[30] Sunehag AL, Man CD, Toffolo G, Haymond MW, Bier DM, Cobelli C. β-Cell function and insulin sensitivity in adolescents from an OGTT. Obesity 2008; 17: 233–9. 19057529 10.1038/oby.2008.496

[31] Bergman RN, Ader M, Bergman, Huecking K, Citters GV. Accurate assessment of β-cell function: the hyperbolic correction. Diabetes 2002; 51: 212–20. 10.2337/diabetes.51.2007.s212 11815482

[32] Retnakaran R, Shen S, Hanley AJ, Vuksan V, Hamilton JK, Zinman B. Hyperbolic relationship between insulin secretion and sensitivity on oral glucose tolerance test. Obesity 2008; 16: 1901–7. 18551118 10.1038/oby.2008.307

[33] Standards of Medical Care in Diabetes-2020. Management of diabetes in pregnancy. Diabetes Care 2020; 43(1)

[34] Nigam A, Sharma S, Varun N, Munjal YP, Prakash A. Comparative analysis of 2-week glycemic profile of healthy versus mild gestational diabetic pregnant women using flash glucose monitoring system: an observational study. BJOG 2019; 126(4): 27–33. 31257712 10.1111/1471-0528.15849

[35] Mustad VA, Huynh DTT, Lōpez-Pedrosa JM, Campoy C, Rueda R. The role of dietary carbohydrates in gestational diabetes. Nutrients 2020; 12(2): 385. 10.3390/nu12020385 PMC7071246 32024026

[36] Chivese T, Norris SA, Levitt NS. High prevalence of cardiovascular risk factors and insulin resistance 6 years after hyperglycemia first detected in pregnancy in Cape Town, South Africa. BMJ Open Diabetes Res Care 2019; 7(1): e000740. 10.1136/bmjdrc-2019-000740 PMC6887495 31803480

[37] Lowe W Jr., Shltens M, Loe LP, Kuang A, Nodzenski M, Talbot O, et al. Association of gestational diabetes with maternal disorders of glucose metabolism and childhood adiposity. JAMA 2018; 20(10): 1005–16. 10.1001/jama.2018.11628 PMC6143108 30208453

